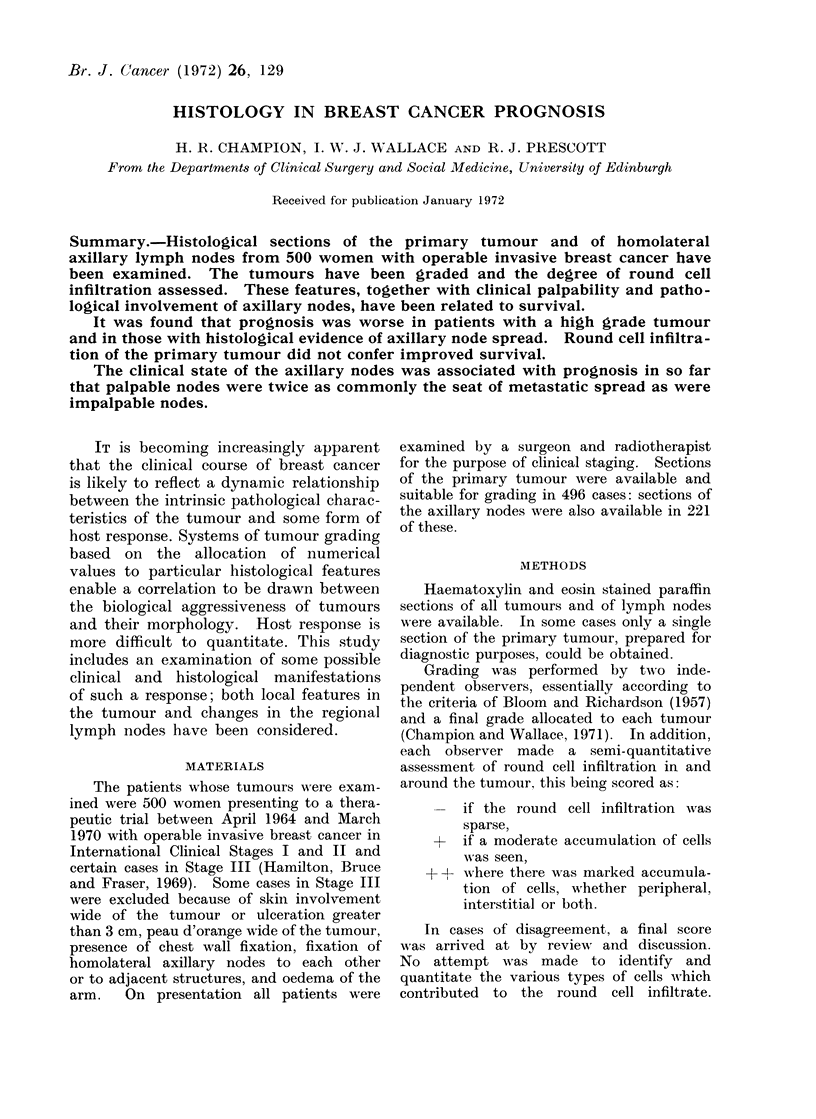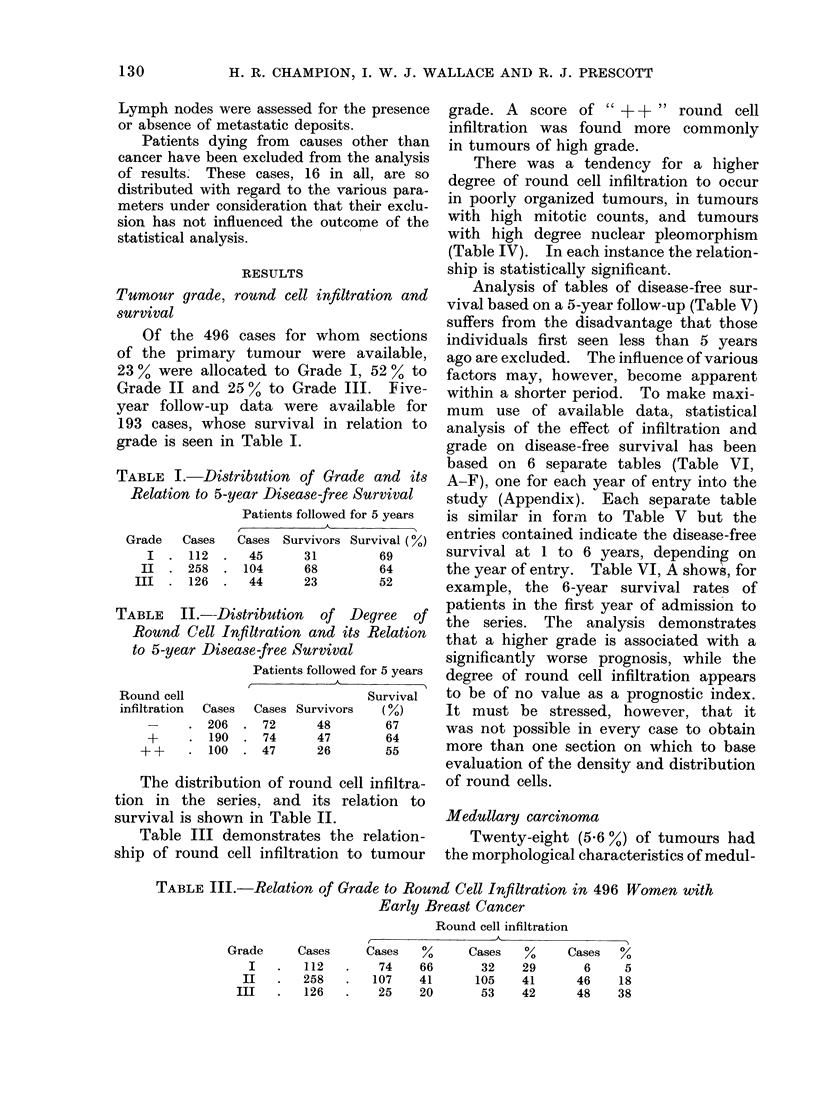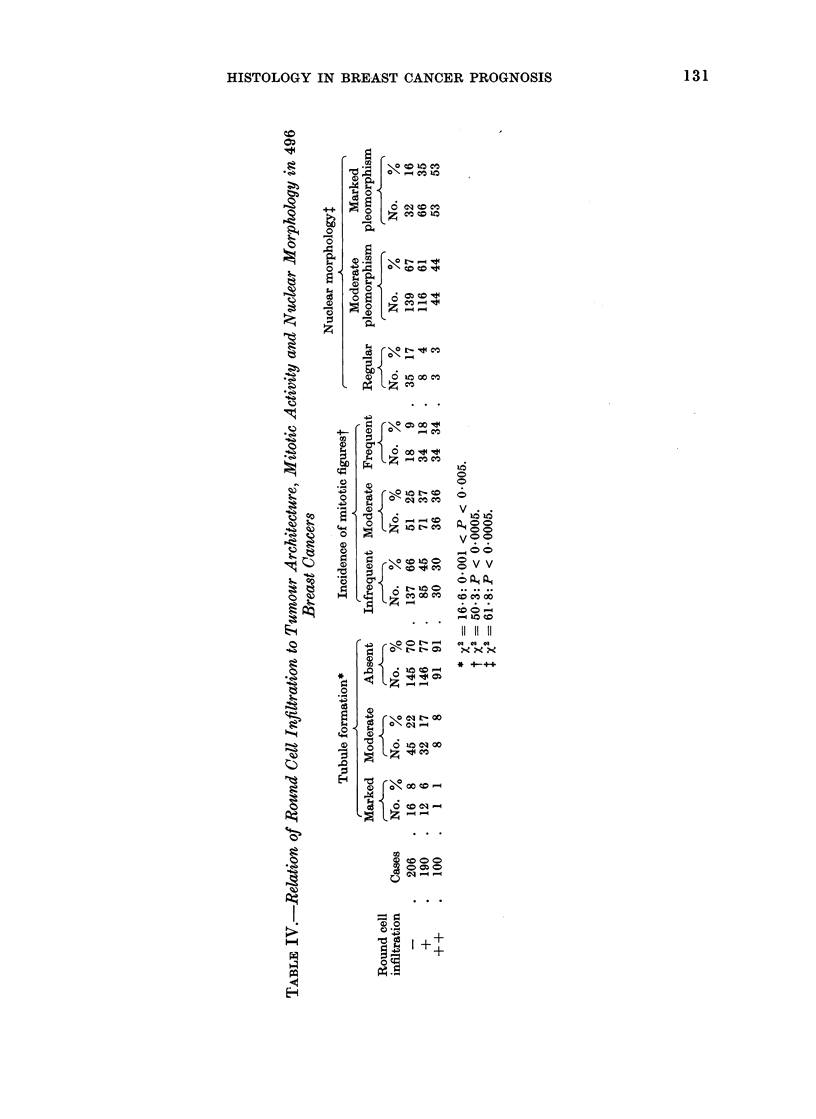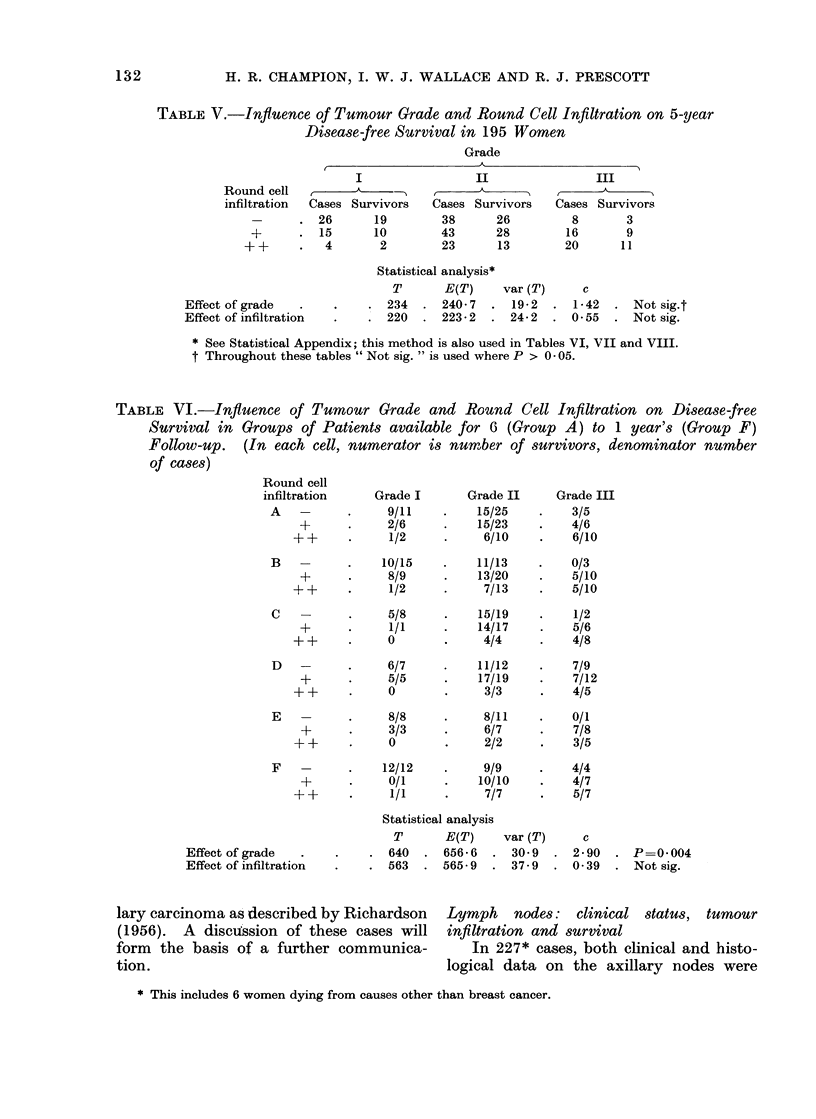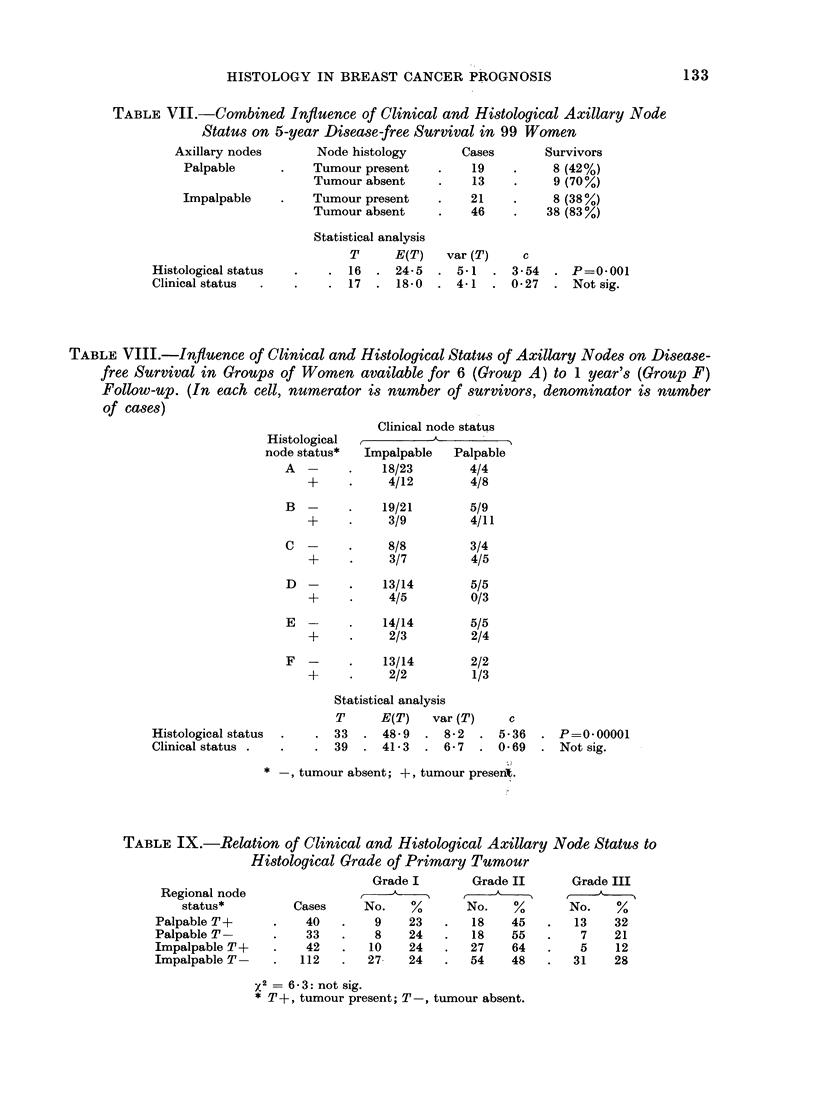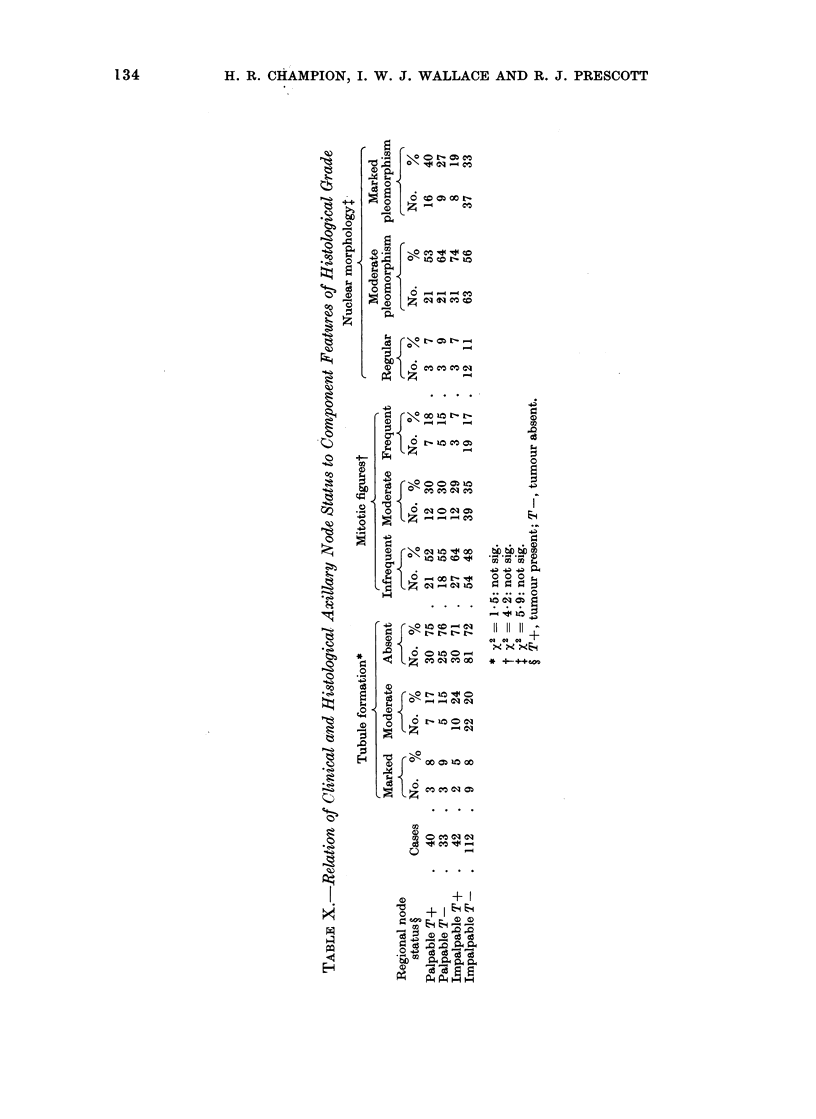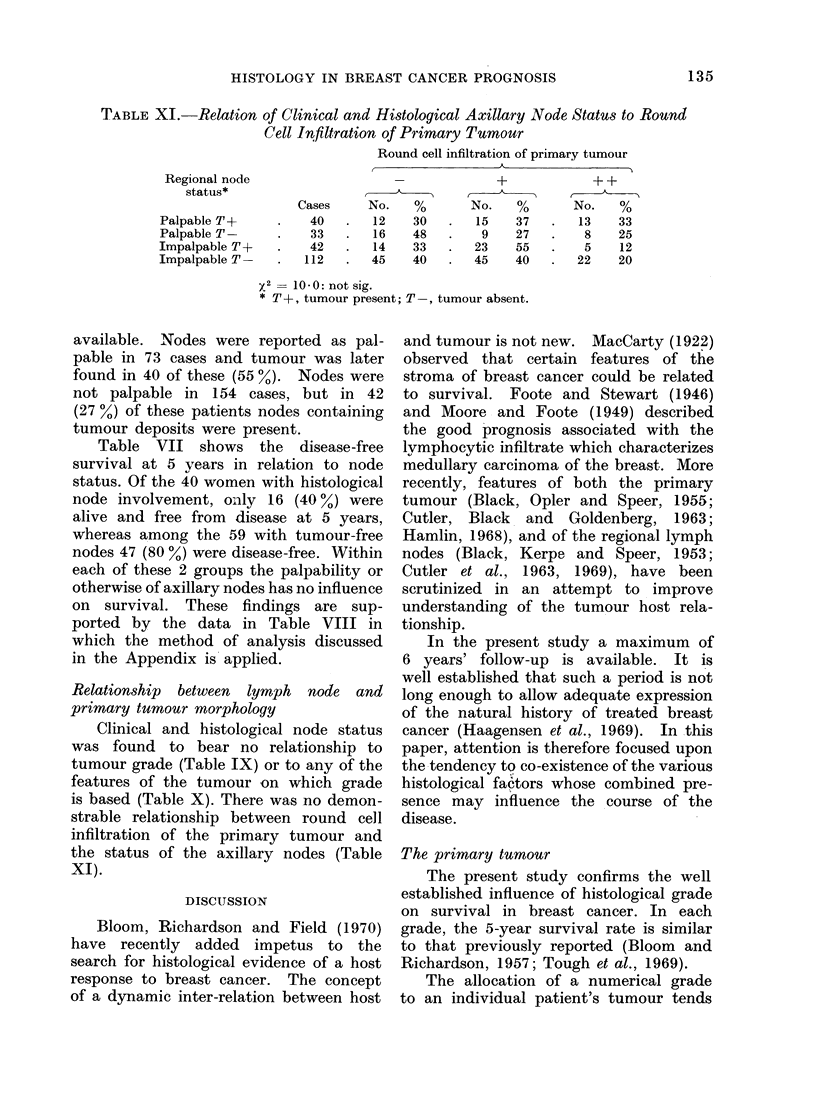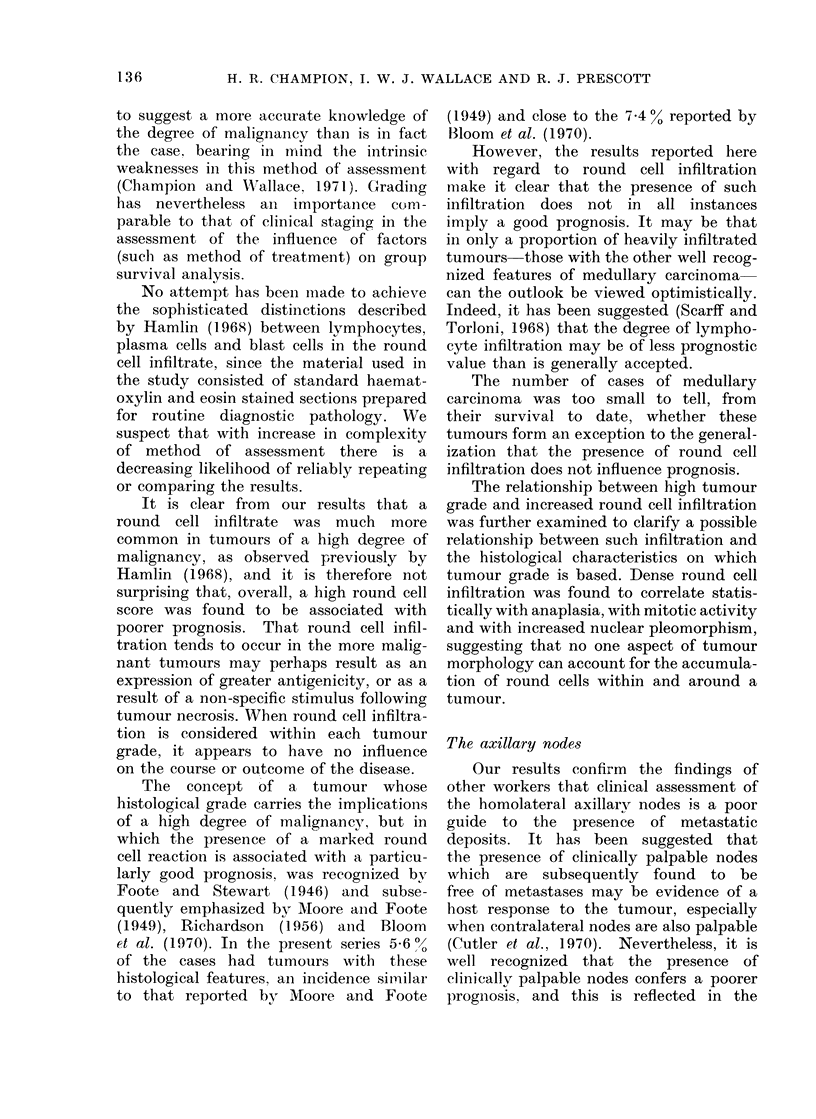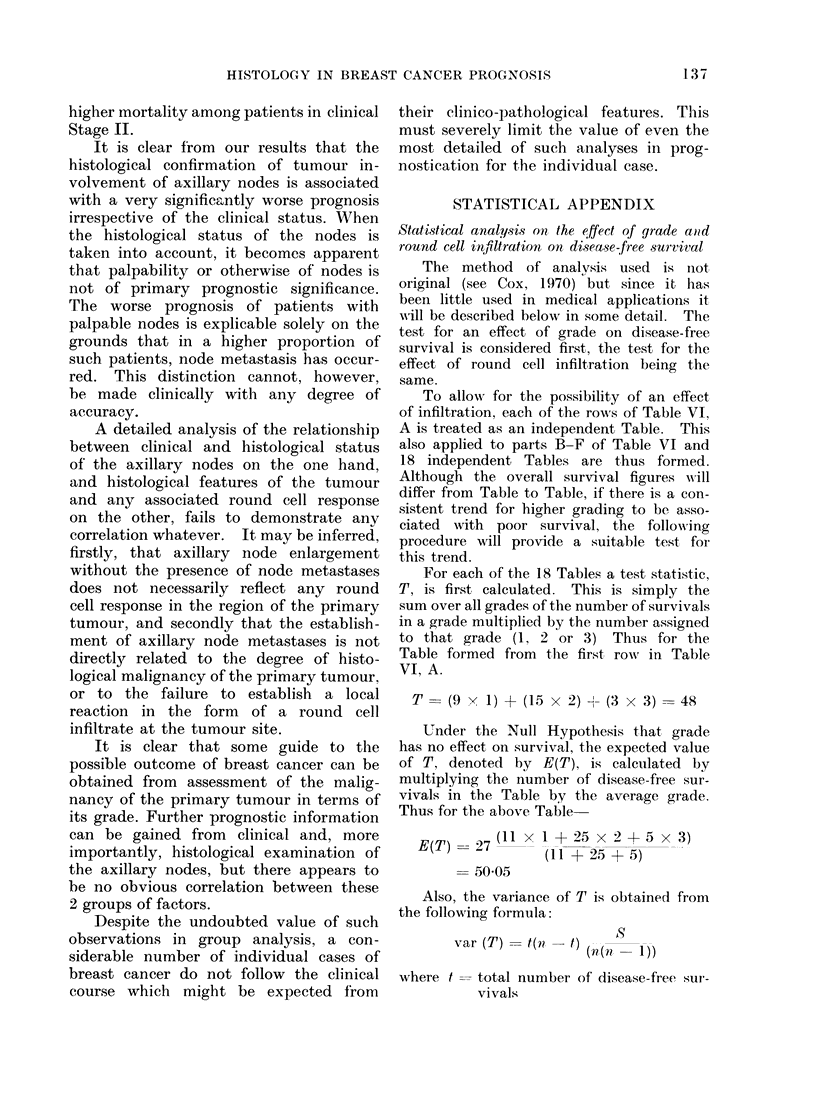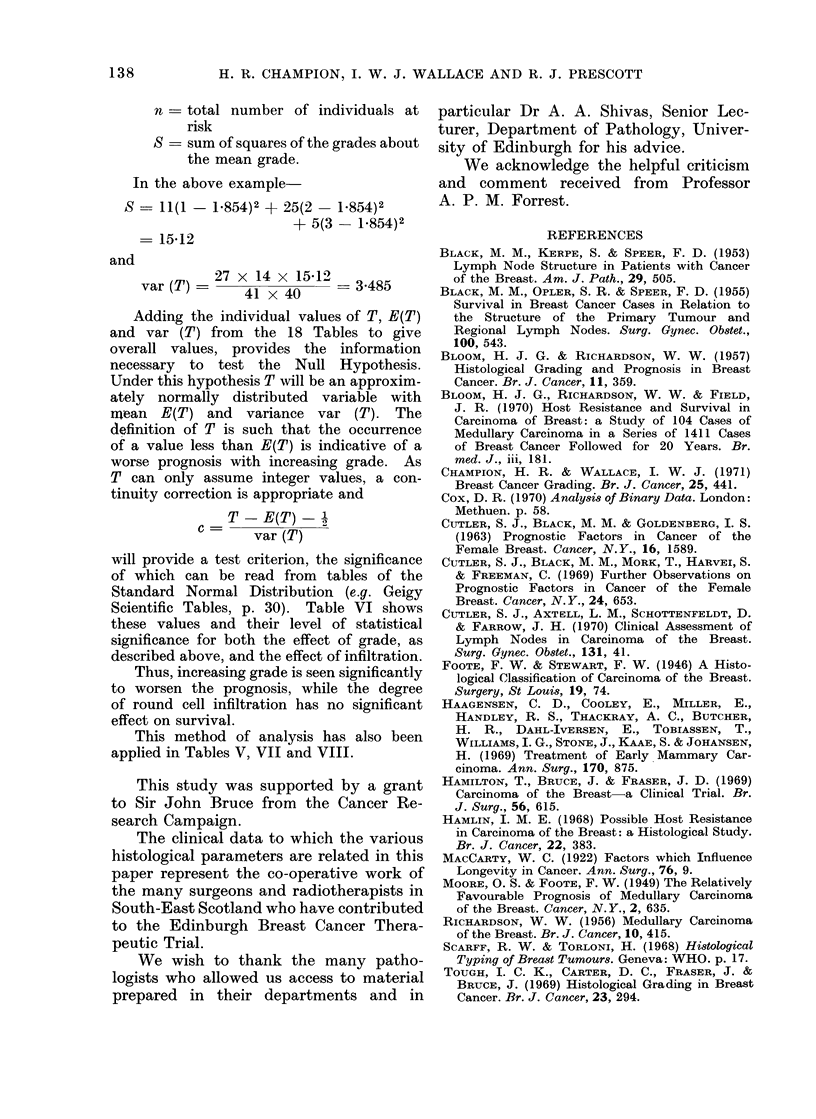# Histology in Breast Cancer Prognosis

**DOI:** 10.1038/bjc.1972.19

**Published:** 1972-04

**Authors:** H. R. Champion, I. W. J. Wallace, R. J. Prescott

## Abstract

Histological sections of the primary tumour and of homolateral axillary lymph nodes from 500 women with operable invasive breast cancer have been examined. The tumours have been graded and the degree of round cell infiltration assessed. These features, together with clinical palpability and pathological involvement of axillary nodes, have been related to survival.

It was found that prognosis was worse in patients with a high grade tumour and in those with histological evidence of axillary node spread. Round cell infiltration of the primary tumour did not confer improved survival.

The clinical state of the axillary nodes was associated with prognosis in so far that palpable nodes were twice as commonly the seat of metastatic spread as were impalpable nodes.


*Br. J. Cancer* (1972) **26**, 129

### HISTOLOGY IN BREAST CANCER PROGNOSIS

H. R. CHAMPION, I. W. J. WALLACE AND R. J. PRESCOTT

*From the Departments of Clinical Surgery and Social Medicine, University of Edinburgh*

Received for publication January 1972

**Summary.—Histological sections of the primary tumour and of homolateral axillary lymph nodes from 500 women with operable invasive breast cancer have been examined. The tumours have been graded and the degree of round cell infiltration assessed. These features, together with clinical palpability and pathological involvement of axillary nodes, have been related to survival.**

**It was found that prognosis was worse in patients with a high grade tumour and in those with histological evidence of axillary node spread. Round cell infiltration of the primary tumour did not confer improved survival.**

**The clinical state of the axillary nodes was associated with prognosis in so far that palpable nodes were twice as commonly the seat of metastatic spread as were impalpable nodes.**

It is becoming increasingly apparent that the clinical course of breast cancer is likely to reflect a dynamic relationship between the intrinsic pathological characteristics of the tumour and some form of host response. Systems of tumour grading based on the allocation of numerical values to particular histological features enable a correlation to be drawn between the biological aggressiveness of tumours and their morphology. Host response is more difficult to quantitate. This study includes an examination of some possible clinical and histological manifestations of such a response; both local features in the tumour and changes in the regional lymph nodes have been considered.

### MATERIALS

The patients whose tumours were examined were 500 women presenting to a therapeutic trial between April 1964 and March 1970 with operable invasive breast cancer in International Clinical Stages I and II and certain cases in Stage III (Hamilton, Bruce and Fraser, 1969). Some cases in Stage III were excluded because of skin involvement wide of the tumour or ulceration greater than 3 cm, peau d'orange wide of the tumour, presence of chest wall fixation, fixation of homolateral axillary nodes to each other or to adjacent structures, and oedema of the arm. On presentation all patients were examined by a surgeon and radiotherapist for the purpose of clinical staging. Sections of the primary tumour were available and suitable for grading in 496 cases: sections of the axillary nodes were also available in 221 of these.

### METHODS

Haematoxylin and eosin stained paraffin sections of all tumours and of lymph nodes were available. In some cases only a single section of the primary tumour, prepared for diagnostic purposes, could be obtained.

Grading was performed by two independent observers, essentially according to the criteria of Bloom and Richardson (1957) and a final grade allocated to each tumour (Champion and Wallace, 1971). In addition, each observer made a semi-quantitative assessment of round cell infiltration in and around the tumour, this being scored as:

− if the round cell infiltration was sparse,
+ if a moderate accumulation of cells was seen,
++ where there was marked accumulation of cells, whether peripheral, interstitial or both.

In cases of disagreement, a final score was arrived at by review and discussion. No attempt was made to identify and quantitate the various types of cells which contributed to the round cell infiltrate.

Lymph nodes were assessed for the presence or absence of metastatic deposits.

Patients dying from causes other than cancer have been excluded from the analysis of results. These cases, 16 in all, are so distributed with regard to the various parameters under consideration that their exclusion has not influenced the outcome of the statistical analysis.

### RESULTS

### *Tumour grade, round cell infiltration and survival*

Of the 496 cases for whom sections of the primary tumour were available, 23% were allocated to Grade I, 52% to Grade II and 25% to Grade III. Five-year follow-up data were available for 193 cases, whose survival in relation to grade is seen in Table I.

TABLE I.—*Distribution of Grade and its Relation to 5-year Disease-free Survival*

| | | Patients followed for 5 years | | |
|---|---|---|---|---|
| Grade | Cases | Cases | Survivors | Survival (%) |
| I | 112 | 45 | 31 | 69 |
| II | 258 | 104 | 68 | 64 |
| III | 126 | 44 | 23 | 52 |

TABLE II.—*Distribution of Degree of Round Cell Infiltration and its Relation to 5-year Disease-free Survival*

| | | Patients followed for 5 years | | |
|---|---|---|---|---|
| Round cell infiltration | Cases | Cases | Survivors | Survival (%) |
| − | 206 | 72 | 48 | 67 |
| + | 190 | 74 | 47 | 64 |
| ++ | 100 | 47 | 26 | 55 |

The distribution of round cell infiltration in the series, and its relation to survival is shown in Table II.

Table III demonstrates the relationship of round cell infiltration to tumour grade. A score of "++" round cell infiltration was found more commonly in tumours of high grade.

There was a tendency for a higher degree of round cell infiltration to occur in poorly organized tumours, in tumours with high mitotic counts, and tumours with high degree nuclear pleomorphism (Table IV). In each instance the relationship is statistically significant.

Analysis of tables of disease-free survival based on a 5-year follow-up (Table V) suffers from the disadvantage that those individuals first seen less than 5 years ago are excluded. The influence of various factors may, however, become apparent within a shorter period. To make maximum use of available data, statistical analysis of the effect of infiltration and grade on disease-free survival has been based on 6 separate tables (Table VI, A–F), one for each year of entry into the study (Appendix). Each separate table is similar in form to Table V but the entries contained indicate the disease-free survival at 1 to 6 years, depending on the year of entry. Table VI, A shows, for example, the 6-year survival rates of patients in the first year of admission to the series. The analysis demonstrates that a higher grade is associated with a significantly worse prognosis, while the degree of round cell infiltration appears to be of no value as a prognostic index. It must be stressed, however, that it was not possible in every case to obtain more than one section on which to base evaluation of the density and distribution of round cells.

### *Medullary carcinoma*

Twenty-eight (5·6%) of tumours had the morphological characteristics of medul-

TABLE III.—*Relation of Grade to Round Cell Infiltration in 496 Women with Early Breast Cancer*

| | | Round cell infiltration | | | | | |
|---|---|---|---|---|---|---|---|
| Grade | Cases | Cases | % | Cases | % | Cases | % |
| I | 112 | 74 | 66 | 32 | 29 | 6 | 5 |
| II | 258 | 107 | 41 | 105 | 41 | 46 | 18 |
| III | 126 | 25 | 20 | 53 | 42 | 48 | 38 |

TABLE IV.—*Relation of Round Cell Infiltration to Tumour Architecture, Mitotic Activity and Nuclear Morphology in* 496 *Breast Cancers*

| Round cell infiltration | Cases | Tubule formation* | | | | | | Incidence of mitotic figures† | | | | | | Nuclear morphology‡ | | | | | |
|---|---|---|---|---|---|---|---|---|---|---|---|---|---|---|---|---|---|---|---|
| | | Marked | | Moderate | | Absent | | Infrequent | | Moderate | | Frequent | | Regular | | Moderate pleomorphism | | Marked pleomorphism | |
| | | No. | % | No. | % | No. | % | No. | % | No. | % | No. | % | No. | % | No. | % | No. | % |
| − | 206 | 16 | 8 | 45 | 22 | 145 | 70 | 137 | 66 | 51 | 25 | 18 | 9 | 35 | 17 | 139 | 67 | 32 | 16 |
| + | 190 | 12 | 6 | 32 | 17 | 146 | 77 | 85 | 45 | 71 | 37 | 34 | 18 | 8 | 4 | 116 | 61 | 66 | 35 |
| ++ | 100 | 1 | 1 | 8 | 8 | 91 | 91 | 30 | 30 | 36 | 36 | 34 | 34 | 3 | 3 | 44 | 44 | 53 | 53 |

* $\chi^2 = 16 \cdot 6: 0 \cdot 001 < P < 0 \cdot 005$.
† $\chi^2 = 50 \cdot 3: P < 0 \cdot 0005$.
‡ $\chi^2 = 61 \cdot 8: P < 0 \cdot 0005$.

TABLE V.—*Influence of Tumour Grade and Round Cell Infiltration on 5-year Disease-free Survival in 195 Women*

| Round cell infiltration | Grade I Cases | Grade I Survivors | Grade II Cases | Grade II Survivors | Grade III Cases | Grade III Survivors |
|---|---|---|---|---|---|---|
| − | 26 | 19 | 38 | 26 | 8 | 3 |
| + | 15 | 10 | 43 | 28 | 16 | 9 |
| ++ | 4 | 2 | 23 | 13 | 20 | 11 |

Statistical analysis*

| | $T$ | $E(T)$ | var ($T$) | $c$ | |
|---|---|---|---|---|---|
| Effect of grade | 234 | 240·7 | 19·2 | 1·42 | Not sig.† |
| Effect of infiltration | 220 | 223·2 | 24·2 | 0·55 | Not sig. |

* See Statistical Appendix; this method is also used in Tables VI, VII and VIII.
† Throughout these tables " Not sig. " is used where $P > 0{\cdot}05$.

TABLE VI.—*Influence of Tumour Grade and Round Cell Infiltration on Disease-free Survival in Groups of Patients available for* 6 (*Group A*) *to* 1 *year's* (*Group F*) *Follow-up.* (*In each cell, numerator is number of survivors, denominator number of cases*)

| Round cell infiltration | | Grade I | Grade II | Grade III |
|---|---|---|---|---|
| A | − | 9/11 | 15/25 | 3/5 |
| | + | 2/6 | 15/23 | 4/6 |
| | ++ | 1/2 | 6/10 | 6/10 |
| B | − | 10/15 | 11/13 | 0/3 |
| | + | 8/9 | 13/20 | 5/10 |
| | ++ | 1/2 | 7/13 | 5/10 |
| C | − | 5/8 | 15/19 | 1/2 |
| | + | 1/1 | 14/17 | 5/6 |
| | ++ | 0 | 4/4 | 4/8 |
| D | − | 6/7 | 11/12 | 7/9 |
| | + | 5/5 | 17/19 | 7/12 |
| | ++ | 0 | 3/3 | 4/5 |
| E | − | 8/8 | 8/11 | 0/1 |
| | + | 3/3 | 6/7 | 7/8 |
| | ++ | 0 | 2/2 | 3/5 |
| F | − | 12/12 | 9/9 | 4/4 |
| | + | 0/1 | 10/10 | 4/7 |
| | ++ | 1/1 | 7/7 | 5/7 |

Statistical analysis

| | $T$ | $E(T)$ | var ($T$) | $c$ | |
|---|---|---|---|---|---|
| Effect of grade | 640 | 656·6 | 30·9 | 2·90 | $P = 0{\cdot}004$ |
| Effect of infiltration | 563 | 565·9 | 37·9 | 0·39 | Not sig. |

lary carcinoma as described by Richardson (1956). A discussion of these cases will form the basis of a further communication.

*Lymph nodes: clinical status, tumour infiltration and survival*

In 227* cases, both clinical and histological data on the axillary nodes were

* This includes 6 women dying from causes other than breast cancer.

TABLE VII.—*Combined Influence of Clinical and Histological Axillary Node Status on 5-year Disease-free Survival in 99 Women*

| Axillary nodes | Node histology | Cases | Survivors |
|---|---|---|---|
| Palpable | Tumour present | 19 | 8 (42%) |
| | Tumour absent | 13 | 9 (70%) |
| Impalpable | Tumour present | 21 | 8 (38%) |
| | Tumour absent | 46 | 38 (83%) |

Statistical analysis

| | $T$ | $E(T)$ | var ($T$) | $c$ | |
|---|---|---|---|---|---|
| Histological status | 16 | 24·5 | 5·1 | 3·54 | $P=0·001$ |
| Clinical status | 17 | 18·0 | 4·1 | 0·27 | Not sig. |

TABLE VIII.—*Influence of Clinical and Histological Status of Axillary Nodes on Disease-free Survival in Groups of Women available for 6 (Group A) to 1 year's (Group F) Follow-up. (In each cell, numerator is number of survivors, denominator is number of cases)*

| Histological node status* | | Clinical node status | |
|---|---|---|---|
| | | Impalpable | Palpable |
| A | − | 18/23 | 4/4 |
| | + | 4/12 | 4/8 |
| B | − | 19/21 | 5/9 |
| | + | 3/9 | 4/11 |
| C | − | 8/8 | 3/4 |
| | + | 3/7 | 4/5 |
| D | − | 13/14 | 5/5 |
| | + | 4/5 | 0/3 |
| E | − | 14/14 | 5/5 |
| | + | 2/3 | 2/4 |
| F | − | 13/14 | 2/2 |
| | + | 2/2 | 1/3 |

Statistical analysis

| | $T$ | $E(T)$ | var ($T$) | $c$ | |
|---|---|---|---|---|---|
| Histological status | 33 | 48·9 | 8·2 | 5·36 | $P=0·00001$ |
| Clinical status | 39 | 41·3 | 6·7 | 0·69 | Not sig. |

* −, tumour absent; +, tumour present.

TABLE IX.—*Relation of Clinical and Histological Axillary Node Status to Histological Grade of Primary Tumour*

| Regional node status* | Cases | Grade I | | Grade II | | Grade III | |
|---|---|---|---|---|---|---|---|
| | | No. | % | No. | % | No. | % |
| Palpable $T+$ | 40 | 9 | 23 | 18 | 45 | 13 | 32 |
| Palpable $T-$ | 33 | 8 | 24 | 18 | 55 | 7 | 21 |
| Impalpable $T+$ | 42 | 10 | 24 | 27 | 64 | 5 | 12 |
| Impalpable $T-$ | 112 | 27 | 24 | 54 | 48 | 31 | 28 |

$\chi^2 = 6·3$: not sig.
* $T+$, tumour present; $T-$, tumour absent.

TABLE X.—*Relation of Clinical and Histological Axillary Node Status to Component Features of Histological Grade*

| | | Tubule formation* | | | | | | Mitotic figures† | | | | | | Nuclear morphology‡ | | | | | |
|---|---|---|---|---|---|---|---|---|---|---|---|---|---|---|---|---|---|---|---|
| | | Marked | | Moderate | | Absent | | Infrequent | | Moderate | | Frequent | | Regular | | Moderate pleomorphism | | Marked pleomorphism | |
| Regional node status§ | Cases | No. | % | No. | % | No. | % | No. | % | No. | % | No. | % | No. | % | No. | % | No. | % |
| Palpable $T+$ | 40 | 3 | 8 | 7 | 17 | 30 | 75 | 21 | 52 | 12 | 30 | 7 | 18 | 3 | 7 | 21 | 53 | 16 | 40 |
| Palpable $T-$ | 33 | 3 | 9 | 5 | 15 | 25 | 76 | 18 | 55 | 10 | 30 | 5 | 15 | 3 | 9 | 21 | 64 | 9 | 27 |
| Impalpable $T+$ | 42 | 2 | 5 | 10 | 24 | 30 | 71 | 27 | 64 | 12 | 29 | 3 | 7 | 3 | 7 | 31 | 74 | 8 | 19 |
| Impalpable $T-$ | 112 | 9 | 8 | 22 | 20 | 81 | 72 | 54 | 48 | 39 | 35 | 19 | 17 | 12 | 11 | 63 | 56 | 37 | 33 |

* $\chi^2 = 1\cdot5$: not sig.

† $\chi^2 = 4\cdot2$: not sig.

‡ $\chi^2 = 5\cdot9$: not sig.

§ $T+$, tumour present; $T-$, tumour absent.

TABLE XI.—*Relation of Clinical and Histological Axillary Node Status to Round Cell Infiltration of Primary Tumour*

| Regional node status* | | Round cell infiltration of primary tumour | | | | | |
|---|---|---|---|---|---|---|---|
| | | − | | + | | ++ | |
| | Cases | No. | % | No. | % | No. | % |
| Palpable $T+$ | 40 | 12 | 30 | 15 | 37 | 13 | 33 |
| Palpable $T-$ | 33 | 16 | 48 | 9 | 27 | 8 | 25 |
| Impalpable $T+$ | 42 | 14 | 33 | 23 | 55 | 5 | 12 |
| Impalpable $T-$ | 112 | 45 | 40 | 45 | 40 | 22 | 20 |

$\chi^2 = 10\cdot0$: not sig.
* $T+$, tumour present; $T-$, tumour absent.

available. Nodes were reported as palpable in 73 cases and tumour was later found in 40 of these (55 %). Nodes were not palpable in 154 cases, but in 42 (27 %) of these patients nodes containing tumour deposits were present.

Table VII shows the disease-free survival at 5 years in relation to node status. Of the 40 women with histological node involvement, only 16 (40 %) were alive and free from disease at 5 years, whereas among the 59 with tumour-free nodes 47 (80 %) were disease-free. Within each of these 2 groups the palpability or otherwise of axillary nodes has no influence on survival. These findings are supported by the data in Table VIII in which the method of analysis discussed in the Appendix is applied.

*Relationship between lymph node and primary tumour morphology*

Clinical and histological node status was found to bear no relationship to tumour grade (Table IX) or to any of the features of the tumour on which grade is based (Table X). There was no demonstrable relationship between round cell infiltration of the primary tumour and the status of the axillary nodes (Table XI).

### DISCUSSION

Bloom, Richardson and Field (1970) have recently added impetus to the search for histological evidence of a host response to breast cancer. The concept of a dynamic inter-relation between host and tumour is not new. MacCarty (1922) observed that certain features of the stroma of breast cancer could be related to survival. Foote and Stewart (1946) and Moore and Foote (1949) described the good prognosis associated with the lymphocytic infiltrate which characterizes medullary carcinoma of the breast. More recently, features of both the primary tumour (Black, Opler and Speer, 1955; Cutler, Black and Goldenberg, 1963; Hamlin, 1968), and of the regional lymph nodes (Black, Kerpe and Speer, 1953; Cutler *et al.*, 1963, 1969), have been scrutinized in an attempt to improve understanding of the tumour host relationship.

In the present study a maximum of 6 years' follow-up is available. It is well established that such a period is not long enough to allow adequate expression of the natural history of treated breast cancer (Haagensen *et al.*, 1969). In this paper, attention is therefore focused upon the tendency to co-existence of the various histological factors whose combined presence may influence the course of the disease.

*The primary tumour*

The present study confirms the well established influence of histological grade on survival in breast cancer. In each grade, the 5-year survival rate is similar to that previously reported (Bloom and Richardson, 1957; Tough *et al.*, 1969).

The allocation of a numerical grade to an individual patient's tumour tends to suggest a more accurate knowledge of the degree of malignancy than is in fact the case, bearing in mind the intrinsic weaknesses in this method of assessment (Champion and Wallace, 1971). Grading has nevertheless an importance comparable to that of clinical staging in the assessment of the influence of factors (such as method of treatment) on group survival analysis.

No attempt has been made to achieve the sophisticated distinctions described by Hamlin (1968) between lymphocytes, plasma cells and blast cells in the round cell infiltrate, since the material used in the study consisted of standard haematoxylin and eosin stained sections prepared for routine diagnostic pathology. We suspect that with increase in complexity of method of assessment there is a decreasing likelihood of reliably repeating or comparing the results.

It is clear from our results that a round cell infiltrate was much more common in tumours of a high degree of malignancy, as observed previously by Hamlin (1968), and it is therefore not surprising that, overall, a high round cell score was found to be associated with poorer prognosis. That round cell infiltration tends to occur in the more malignant tumours may perhaps result as an expression of greater antigenicity, or as a result of a non-specific stimulus following tumour necrosis. When round cell infiltration is considered within each tumour grade, it appears to have no influence on the course or outcome of the disease.

The concept of a tumour whose histological grade carries the implications of a high degree of malignancy, but in which the presence of a marked round cell reaction is associated with a particularly good prognosis, was recognized by Foote and Stewart (1946) and subsequently emphasized by Moore and Foote (1949), Richardson (1956) and Bloom *et al.* (1970). In the present series 5·6 % of the cases had tumours with these histological features, an incidence similar to that reported by Moore and Foote (1949) and close to the 7·4 % reported by Bloom *et al.* (1970).

However, the results reported here with regard to round cell infiltration make it clear that the presence of such infiltration does not in all instances imply a good prognosis. It may be that in only a proportion of heavily infiltrated tumours—those with the other well recognized features of medullary carcinoma—can the outlook be viewed optimistically. Indeed, it has been suggested (Scarff and Torloni, 1968) that the degree of lymphocyte infiltration may be of less prognostic value than is generally accepted.

The number of cases of medullary carcinoma was too small to tell, from their survival to date, whether these tumours form an exception to the generalization that the presence of round cell infiltration does not influence prognosis.

The relationship between high tumour grade and increased round cell infiltration was further examined to clarify a possible relationship between such infiltration and the histological characteristics on which tumour grade is based. Dense round cell infiltration was found to correlate statistically with anaplasia, with mitotic activity and with increased nuclear pleomorphism, suggesting that no one aspect of tumour morphology can account for the accumulation of round cells within and around a tumour.

### *The axillary nodes*

Our results confirm the findings of other workers that clinical assessment of the homolateral axillary nodes is a poor guide to the presence of metastatic deposits. It has been suggested that the presence of clinically palpable nodes which are subsequently found to be free of metastases may be evidence of a host response to the tumour, especially when contralateral nodes are also palpable (Cutler *et al.*, 1970). Nevertheless, it is well recognized that the presence of clinically palpable nodes confers a poorer prognosis, and this is reflected in the higher mortality among patients in clinical Stage II.

It is clear from our results that the histological confirmation of tumour involvement of axillary nodes is associated with a very significantly worse prognosis irrespective of the clinical status. When the histological status of the nodes is taken into account, it becomes apparent that palpability or otherwise of nodes is not of primary prognostic significance. The worse prognosis of patients with palpable nodes is explicable solely on the grounds that in a higher proportion of such patients, node metastasis has occurred. This distinction cannot, however, be made clinically with any degree of accuracy.

A detailed analysis of the relationship between clinical and histological status of the axillary nodes on the one hand, and histological features of the tumour and any associated round cell response on the other, fails to demonstrate any correlation whatever. It may be inferred, firstly, that axillary node enlargement without the presence of node metastases does not necessarily reflect any round cell response in the region of the primary tumour, and secondly that the establishment of axillary node metastases is not directly related to the degree of histological malignancy of the primary tumour, or to the failure to establish a local reaction in the form of a round cell infiltrate at the tumour site.

It is clear that some guide to the possible outcome of breast cancer can be obtained from assessment of the malignancy of the primary tumour in terms of its grade. Further prognostic information can be gained from clinical and, more importantly, histological examination of the axillary nodes, but there appears to be no obvious correlation between these 2 groups of factors.

Despite the undoubted value of such observations in group analysis, a considerable number of individual cases of breast cancer do not follow the clinical course which might be expected from their clinico-pathological features. This must severely limit the value of even the most detailed of such analyses in prognostication for the individual case.

### STATISTICAL APPENDIX

*Statistical analysis on the effect of grade and round cell infiltration on disease-free survival*

The method of analysis used is not original (see Cox, 1970) but since it has been little used in medical applications it will be described below in some detail. The test for an effect of grade on disease-free survival is considered first, the test for the effect of round cell infiltration being the same.

To allow for the possibility of an effect of infiltration, each of the rows of Table VI, A is treated as an independent Table. This also applied to parts B–F of Table VI and 18 independent Tables are thus formed. Although the overall survival figures will differ from Table to Table, if there is a consistent trend for higher grading to be associated with poor survival, the following procedure will provide a suitable test for this trend.

For each of the 18 Tables a test statistic, $T$, is first calculated. This is simply the sum over all grades of the number of survivals in a grade multiplied by the number assigned to that grade (1, 2 or 3) Thus for the Table formed from the first row in Table VI, A.

$$T = (9 \times 1) + (15 \times 2) + (3 \times 3) = 48$$

Under the Null Hypothesis that grade has no effect on survival, the expected value of $T$, denoted by $E(T)$, is calculated by multiplying the number of disease-free survivals in the Table by the average grade. Thus for the above Table—

$$E(T) = 27 \frac{(11 \times 1 + 25 \times 2 + 5 \times 3)}{(11 + 25 + 5)}$$

$$= 50{\cdot}05$$

Also, the variance of $T$ is obtained from the following formula:

$$\text{var}\ (T) = t(n - t) \frac{S}{(n(n - 1))}$$

where $t$ = total number of disease-free survivals

$n$ = total number of individuals at risk
$S$ = sum of squares of the grades about the mean grade.

In the above example—

$$S = 11(1 - 1{\cdot}854)^2 + 25(2 - 1{\cdot}854)^2 + 5(3 - 1{\cdot}854)^2 = 15{\cdot}12$$

and

$$\text{var}\ (T) = \frac{27 \times 14 \times 15{\cdot}12}{41 \times 40} = 3{\cdot}485$$

Adding the individual values of $T$, $E(T)$ and var ($T$) from the 18 Tables to give overall values, provides the information necessary to test the Null Hypothesis. Under this hypothesis $T$ will be an approximately normally distributed variable with mean $E(T)$ and variance var ($T$). The definition of $T$ is such that the occurrence of a value less than $E(T)$ is indicative of a worse prognosis with increasing grade. As $T$ can only assume integer values, a continuity correction is appropriate and

$$c = \frac{T - E(T) - \frac{1}{2}}{\text{var}\ (T)}$$

will provide a test criterion, the significance of which can be read from tables of the Standard Normal Distribution (*e.g.* Geigy Scientific Tables, p. 30). Table VI shows these values and their level of statistical significance for both the effect of grade, as described above, and the effect of infiltration.

Thus, increasing grade is seen significantly to worsen the prognosis, while the degree of round cell infiltration has no significant effect on survival.

This method of analysis has also been applied in Tables V, VII and VIII.

This study was supported by a grant to Sir John Bruce from the Cancer Research Campaign.

The clinical data to which the various histological parameters are related in this paper represent the co-operative work of the many surgeons and radiotherapists in South-East Scotland who have contributed to the Edinburgh Breast Cancer Therapeutic Trial.

We wish to thank the many pathologists who allowed us access to material prepared in their departments and in particular Dr A. A. Shivas, Senior Lecturer, Department of Pathology, University of Edinburgh for his advice.

We acknowledge the helpful criticism and comment received from Professor A. P. M. Forrest.

### REFERENCES

Black, M. M., Kerpe, S. & Speer, F. D. (1953) Lymph Node Structure in Patients with Cancer of the Breast. *Am. J. Path.*, **29**, 505.

Black, M. M., Opler, S. R. & Speer, F. D. (1955) Survival in Breast Cancer Cases in Relation to the Structure of the Primary Tumour and Regional Lymph Nodes. *Surg. Gynec. Obstet.*, **100**, 543.

Bloom, H. J. G. & Richardson, W. W. (1957) Histological Grading and Prognosis in Breast Cancer. *Br. J. Cancer*, **11**, 359.

Bloom, H. J. G., Richardson, W. W. & Field, J. R. (1970) Host Resistance and Survival in Carcinoma of Breast: a Study of 104 Cases of Medullary Carcinoma in a Series of 1411 Cases of Breast Cancer Followed for 20 Years. *Br. med. J.*, iii, 181.

Champion, H. R. & Wallace, I. W. J. (1971) Breast Cancer Grading. *Br. J. Cancer*, **25**, 441.

Cox, D. R. (1970) *Analysis of Binary Data.* London: Methuen. p. 58.

Cutler, S. J., Black, M. M. & Goldenberg, I. S. (1963) Prognostic Factors in Cancer of the Female Breast. *Cancer, N.Y.*, **16**, 1589.

Cutler, S. J., Black, M. M., Mork, T., Harvei, S. & Freeman, C. (1969) Further Observations on Prognostic Factors in Cancer of the Female Breast. *Cancer, N.Y.*, **24**, 653.

Cutler, S. J., Axtell, L. M., Schottenfeldt, D. & Farrow, J. H. (1970) Clinical Assessment of Lymph Nodes in Carcinoma of the Breast. *Surg. Gynec. Obstet.*, **131**, 41.

Foote, F. W. & Stewart, F. W. (1946) A Histological Classification of Carcinoma of the Breast. *Surgery, St Louis*, **19**, 74.

Haagensen, C. D., Cooley, E., Miller, E., Handley, R. S., Thackray, A. C., Butcher, H. R., Dahl-Iversen, E., Tobiassen, T., Williams, I. G., Stone, J., Kaae, S. & Johansen, H. (1969) Treatment of Early Mammary Carcinoma. *Ann. Surg.*, **170**, 875.

Hamilton, T., Bruce, J. & Fraser, J. D. (1969) Carcinoma of the Breast—a Clinical Trial. *Br. J. Surg.*, **56**, 615.

Hamlin, I. M. E. (1968) Possible Host Resistance in Carcinoma of the Breast: a Histological Study. *Br. J. Cancer*, **22**, 383.

MacCarty, W. C. (1922) Factors which Influence Longevity in Cancer. *Ann. Surg.*, **76**, 9.

Moore, O. S. & Foote, F. W. (1949) The Relatively Favourable Prognosis of Medullary Carcinoma of the Breast. *Cancer, N.Y.*, **2**, 635.

Richardson, W. W. (1956) Medullary Carcinoma of the Breast. *Br. J. Cancer*, **10**, 415.

Scarff, R. W. & Torloni, H. (1968) *Histological Typing of Breast Tumours.* Geneva: WHO. p. 17.

Tough, I. C. K., Carter, D. C., Fraser, J. & Bruce, J. (1969) Histological Grading in Breast Cancer. *Br. J. Cancer*, **23**, 294.